# TIMP-1 Modulation Correlates with KRAS Dependency and EMT Induction in NSCLC

**DOI:** 10.3390/cells14181413

**Published:** 2025-09-10

**Authors:** Ilamathi M-Thirusenthilarasan, Pankaj Ahluwalia, Nithyananda Thorenoor, Sampa Ghoshal-Gupta, Byung Rho Lee, Bilal Siddiqui, Ravindra Kolhe, Amyn M. Rojiani, Mumtaz V. Rojiani

**Affiliations:** 1 Department of Pathology, Penn State College of Medicine, Hershey, PA 17033, USA; ithirusenthilarasan@pennstatehealth.psu.edu (I.M.-T.); nithyananda.thorenoor@gmail.com (N.T.); bsiddiqui@pennstatehealth.psu.edu (B.S.); arojiani@pennstatehealth.psu.edu (A.M.R.); 2 Department of Pathology, Medical College of Georgia, Augusta University, Augusta, GA 30912, USA; pahluwalia@augusta.edu (P.A.); sampa.guptaghoshal@gmail.com (S.G.-G.); domadoma222@gmail.com (B.R.L.); rkolhe@augusta.edu (R.K.); 3 Penn State Cancer Institute, Penn State College of Medicine, Hershey, PA 17033, USA; 4 Departments of Neuroscience and Experimental Therapeutics, Penn State College of Medicine, Hershey, PA 17033, USA

**Keywords:** KRAS, non-small cell lung carcinoma, TIMP-1, epithelial–mesenchymal transition, oncogene addiction

## Abstract

Kirsten rat sarcoma viral oncogene homolog (KRAS) is one of the most frequently mutated genes in human cancer, including non-small cell lung carcinoma (NSCLC). Sustained expression of KRAS is required for survival in KRAS-dependent tumors. KRAS tumors can become independent upon bypassing this addiction. Tissue inhibitor of metalloproteinase-1 (TIMP-1) exhibits a range of novel functions in addition to its initially recognized activity as a physiological inhibitor of matrix metalloproteinases (MMPs). It has repeatedly been associated with cancer progression and poor prognosis in multiple cancers. This study investigates the relationship between TIMP-1 modulation and KRAS dependency in NSCLC. We found an inverse expression of KRAS and TIMP-1 in NSCLC lines. Modulating TIMP-1 levels altered KRAS expression and affected KRAS-dependency features. Overexpression of TIMP-1 decreases the KRAS levels in dependent cells and knocking-down TIMP-1 increases KRAS levels in independent cells with concomitant change in RAS-GTP levels. TIMP-1 modulation influenced apoptosis upon KRAS ablation, with TIMP-1 overexpression decreasing apoptosis in dependent cells and TIMP-1 knockdown increasing it in independent cells. Bioinformatic analysis depicted variant-specific perturbations between KRAS and TIMP-1 expression. Furthermore, EMT marker expression was altered upon TIMP-1 modulation, suggesting the role of TIMP-1 in EMT induction in KRAS-independent cells. These findings emphasize the intricate relationship between TIMP-1 and KRAS in NSCLC, shedding light on potential mechanisms underlying tumor behavior and response to therapy.

## 1. Introduction

As a leading cause of cancer-related deaths worldwide, lung cancer remains a significant health concern. NSCLC, with its histological and molecular subtypes, presents a truly heterogenous entity with dismal outcomes.

The most frequently mutated genes in lung adenocarcinomas are *KRAS* and Epidermal Growth Factor Receptor (*EGFR*), molecules in a common signaling pathway [1]. Targeted therapy using receptor tyrosine kinase inhibitors has shown dramatic positive clinical response in EGFR mutated tumors [2,3]. However, KRAS, which is mutated in 30% of lung adenocarcinomas, has long been defined as ‘undruggable’. Hence efforts have been geared to targeting effectors downstream of KRAS [4]. Although there are clinical trials for drugs targeting G12C mutations of *KRAS* [5], other mutations are still undruggable and chemoresistance development remains an ongoing challenge. Approximately 25% of lung adenocarcinomas carry oncogenic mutations in the *KRAS* oncogene, leading to the activation of downstream survival pathways [6,7].

The concept of ‘oncogene addiction’ was defined two decades ago [8], proposing the apparent dependency of some cancers on a single gene or few genes to maintain the malignant phenotype. Over the years oncogenic addiction has been documented for KRAS mutants, whereby lung and pancreatic adenocarcinoma cell lines harboring *KRAS* mutations have been classified as KRAS-dependent or KRAS-independent on the basis of *KRAS* shRNA knockdown resulting in apoptosis [9]. KRAS-dependent cells exhibit a more epithelial-like morphology as well as the genomic amplification of KRAS. KRAS-independent cell lines appear to bypass KRAS and have undergone epithelial to mesenchymal transition (EMT) [9]. Although the process of EMT occurs under physiological conditions of gastrulation where primitive epithelia undergo EMT to facilitate development and this is referred to as Type 1 EMT. Type 2 EMT occurs during organ fibrosis as well as wound healing [10]. However, it is Type 3 EMT that plays a crucial role in cancer progression [10].

Tissue inhibitor of metalloproteinase-1 (TIMP-1) is one of the four endogenous inhibitors of enzymes responsible for extracellular matrix turnover [11]. Classically regarded as an inhibitor of tumor progression, TIMP-1 upregulation has consistently been associated with poor prognosis in all cancers across the board [12]. TIMP1′s role in cancer has been found to be MMP-independent affecting proliferation, apoptosis, angiogenesis and chemoresistance. The most well-documented function of TIMP1 is its inhibition of apoptosis, as shown by multiple studies, including ours [13,14,15,16]. In the last few years, this anti-apoptotic function has been translated into a role for TIMP1 in chemoresistance by us as well as others [17,18,19].

In recent years, TIMP-1′s role in EMT has surfaced, wherein TIMP-1 overexpression induces EMT phenotypes in fibroblasts, MDCK cells, and breast epithelial cells [20,21,22]. Additionally, it has been shown that RAS-induced EMT upregulates TIMP-1 [23]. Over the years, we have studied the novel functions of TIMP-1 in NSCLC [13,17,24,25,26]. All the NSCLC cell lines utilized in our studies have KRAS mutations, with some expressing high endogenous levels of TIMP-1 and others expressing low levels. We therefore undertook this study to identify functional relationships between KRAS and TIMP-1. We found that KRAS-independent cells express high levels of TIMP-1. We then knocked down and overexpressed TIMP-1 in independent and dependent cell lines, respectively. Subjecting them to KRAS ablation/apoptosis, EMT marker analysis, functional assays for tumorigenesis, downstream effector pathways and bioinformatics, we show that modulating their TIMP-1 levels caused alteration in the KRAS -dependency features of NSCLC cell lines affecting apoptosis, EMT marker expression, tumorigenicity, and signaling.

## 2. Methods

### 2.1. Cell Lines and Culture Conditions

Human NSCLC cell lines (H2009, H441, H2122, A549, H460, and SKLU-1) were purchased from American Type Culture Collection (ATCC, Manassas, VA, USA). All the other cell clones, encoding non-target-scrambled shRNA (NT), TIMP-1-specific activation particles (OE), or TIMP-1-specific knockdown shRNA (KD) sequences characterized in our previous studies were used. H2009, H441, H2122, and H460 and their clones were grown in RPMI-1640 medium; A549 and its clones were cultured in F-12K medium; and SKLU-1 and its clones were grown in DMEM as per ATCC recommendations. To generate overexpressing and knockdown clones of TIMP-1, we used TIMP1-activating Lentiviral particles (TIMP-1 OE clone) (Santa Cruz, Dallas, TX, USA); shRNA Lentiviral Particles (Sigma-Aldrich, Burlington, MA, USA) for TIMP-1 KD and NT clones, as described in previous studies [24]. All human cell lines were authenticated using STR (or SNP) profiling within the last three years and were mycoplasma-free.

### 2.2. Reverse Transcription Quantitative Real-Time PCR (RT-qPCR)

RT-qPCR analysis was performed as previously described to titrate the expression level of *TIMP-1*, *KRAS*, and *Zinc Finger E-Box Binding Homeobox (ZEB)2*. Briefly, total RNA was extracted and purified using TRIzol method with the PureLink RNA Mini Kit (Invitrogen, Carlsbad, CA, USA), and 1 μg RNA was reverse transcribed using the RT^2^ First Strand Kit for cDNA synthesis (Qiagen). The amplification of cDNA was performed by real-time PCR on the Quant Studio 3 Real-Time PCR System (Applied Biosystems, Thermo Fisher Scientific, Waltham, MA, USA) using the RT^2^ qPCR Primer Assay (specific for *TIMP1*, *KRAS*, *ZEB2*, and *Glyceraldehyde-3-phosphate dehydrogenase* (*GAPDH*) and the RT^2^ SYBR Green Mastermix (Qiagen, Germantown, MD, USA) with the following settings: 95 °C for 10 min, followed by 40 cycles of amplification at 95 °C for 15 s and 60 °C for 1 min. The 2^−∆∆Ct^ method was utilized for data analysis, with normalization performed against the housekeeping gene. Data are reported as mean values with standard deviations (±SD). Gene expression was determined in triplicate in each reaction for at least three independent experiments.

### 2.3. Western Blotting

Whole-cell proteins were extracted using 1X cell lysis buffer (Cell Signaling Technology, Danvers, MA, USA) on ice supplemented with PMSF (1 mM final concentration) and phosphatase inhibitors (1X Roche- phosphoSTOP^TM^) and then briefly sonicated. Following protein quantitation (BCA kit, Thermoscientific, Waltham, MA, USA) 20–50 µg were separated on 4–15% SDS-PAGE (Bio-Rad, Hercules, CA, USA) and transferred to PVDF membranes (MilliporeSigma, Burlington, MA, USA) and blocked in 5% Blotting-Grade Blocker (Bio-Rad Hercules, CA, USA) in Tris-buffered saline with 0.05% Tween-20 (TBS-T). The membranes were incubated overnight (4 °C) with primary antibodies against TIMP-1 (5 µg/mL), KRAS (2 µg/mL), PARP (1:1000 dilution), p/t-ERK (1:1000), p/t-p90RSK (1:1000), p/t AKT (Thr 308) (1:1000), p/tYAP1 (1:1000), p/tBAD (ser 112) (1:1000), and β-actin (1:1000). Horseradish peroxidase-conjugated secondary antibodies (1:3000) were applied to the membranes, and then the protein expression levels were detected using the enhanced chemiluminescence system (SuperSignal^TM^ West Femto, Thermo Scientific, Waltham, MA, USA). Data were quantified by densitometry using Image Lab (version 6.1, BioRad, Hercules, CA USA) and normalized to β-actin as the loading control.

### 2.4. RAS Activity Assay

RAS activation was measured using a Ras GTPase chemiluminescent ELISA kit (ab134640, Abcam, Cambridge, UK) as per the manufacturer’s instructions. Briefly, the chosen NSCLC cells were cultured for 48 h with 80% confluency. The whole cell proteins were extracted using the reagents and protocols as described by the manufacturer. The protein content was measured using BCA assay kit (Thermo Scientific, Waltham MA, USA). The ELISA assay was performed immediately in the glutathione pre-coated plate provided. It was then coated with Raf-RBD protein (fused to GST) and incubated at 4 °C. 15 μg (in 50 μL) of protein samples were loaded in each well and the EGF-treated Hela extracts were used as positive control (25 μg). After incubation and washing of unbound proteins, the primary antibody (1:500) was added and incubated at room temperature for 1 h; followed by secondary antibody (1:5000) incubation. The chemiluminescent substrate reagents were added and read using a Spectra Max M3 plate reader (Molecular Devices, San Jose, CA, USA) within 15 min. 

### 2.5. siRNA Transfection

*KRAS* was silenced with duplex siRNA targeting *KRAS* (Santa Cruz Biotech). The cells were also transfected with scrambled siRNA (Santa Cruz Biotech, Dallas, TX, USA) as the control. Transfection was carried out using siRNA Transfection reagent (Santa Cruz, Dallas, TX, USA). NSCLC cells (parental, OE, and KD clones) were plated in 60 mm dishes and transfected with 6 μg siRNA. Finally, the cultures were harvested after 72 h post-transfection for (KRAS and PARP) expression analyses. After optimization and confirmation of successful transfection, the same protocol was carried out in a 4-well chambered slide. After 72 h of transfection, the cells were fixed and TUNEL assay was carried out immediately.

### 2.6. A549 KD Cells with Rescue Plasmid

A549 KD clones were transfected with the rescue plasmid to revert the knockdown effect as described earlier [25]. The rescue plasmid was constructed to contain a CMV promoter, the full-length cDNA of TIMP-1 resistant to shRNA, and a Neomycin resistant gene. The shRNA binding site of TIMP-1 cDNA was mutated using PCR primers to create a shRNA-resistant TIMP-1 cDNA. The mutated *TIMP1* expression cassette along with the selection marker was amplified by PCR method from *TIMP1*-expressing plasmid pCMV-TIMP-1 with the following primers (5′ to 3′):

(1) CAAGCTTAAGCGGCCGCTAGTTATTAATAGTAATCAATTACG;
(2) GTCGGAATTGCAaAAcGCaGTCTGTGGGTG;
(3) CACCCACAGACtGCgTTtTGCAATTCCGAC;
(4) GTCCCTCGACGAATTCCTGGGACCGAACCCCGCGT.

The mutation sites are denoted by lower case letters. The amplified DNA fragments were subcloned into NotI and EcoRI site of pLentiLox3.7 plasmid to create pLL-antiKD-TIMP-1 and the mutated *TIMP1* was transfected in *TIMP1* knockdown cells. The sequence was confirmed by DNA sequencing (MC labs, South San Francisco, CA, USA). These PLL-anti-KD-TIMP-1 cells were also transfected with siRNA-KRAS and post-transfection PARP expression, and TUNEL assays were performed.

### 2.7. TUNEL Assay

The KRAS-silenced cells were fixed in the slide chambers using 4% PFA and TUNEL assay was performed using Click-iT^TM^ Plus TUNEL Assay Kit (Invitrogen, Carlsbad, CA, USA), as described by the manufacturer. This is an in-situ apoptosis detection method with Alexa Fluor 594 dye. The nuclei were counter-stained with 1X Hoechst 33,342 stain. The fluorescent signals obtained were detected using ECHO Revolve 4, Hybrid Fluorescence Microscope (10× magnification). The number of TUNEL nuclei (Red spots) per 100 nuclei were counted in three different fields and plotted in the graph.

### 2.8. Anchorage-Independent Soft Agar Assay 

A heterogeneous mixture of 5 × 10^3^ cells in 0.5 mL of 0.6% agar in medium was layered on top of 0.5 mL of 0.6% agar in a 24-well plate. The cells were fed once a week by adding complete media on the top agar. After 22 days, the colonies formed were noted and their images captured with a microscope at 5× magnification.

### 2.9. Wound Assay

Cells were grown until they reached 90–95% confluence in 6-well plates. A sterile 200 μL pipette tip was used to make a cross-shaped wound. Then, the cells were gently rinsed with PBS. Subsequently the cells that migrated to the wounded area were imaged at 0, 24, 48, 72, and 96 h. The percentage of wound closure was also evaluated using ImageJ version 1.54p.

### 2.10. Spheroid Formation Assay

The ultra-low adhesion 24-well plates were coated with 200 µL of Matrigel and incubated at 37 °C. The NSCLC cells collected in their exponential growth phase were mixed with equal volume of media and Matrigel (2000 cells suspended in 25 μL media and 25 μL Matrigel for 1 well). This mixture was added gently to the Matrigel-coated wells and incubated for an hour at 37 °C. Warm complete media was suspended on top of the cells and left undisturbed. The cells were continuously fed between 2–3 days. After 10 days, the spheres formed were imaged in a microscope at (5× magnification).

### 2.11. Immunofluorescence Assay

Cells were seeded on a 4-well chambered glass slide (BD Biosciences, San Jose, CA, USA) and were grown as a monolayer for 48 h. These cells were then fixed in 4% paraformaldehyde for 10 min at room temperature, then washed 3 times with PBS. Cells were permeabilized using 0.1% Triton X-100 in PBS for 15 min and blocked with 5% goat serum in PBS with for 1 h. Cells were incubated with E-cadherin, YAP1 and pYAP1 primary antibodies (1:200 diluted) in blocking buffer overnight at 4 °C. After washing three times with PBS, fluorescence-conjugated secondary antibodies Alexa fluor 594 (1:500 diluted) were applied correspondingly. Isotype-specific IgG was used as the negative control. Nuclei were stained with 1X Hoechst 33,342 dye and visualized using a fluorescence microscope. To visualize the F-actin in the cells, after permeabilization of the fixed cells, 100 nM Rhodamine Phalloidin (Cytoskeleton Inc., Denver, CO, USA) was added and incubated in a humid chamber at room temperature in the dark for 30 min. Before adding Rhodamine stain, anti-TIMP-1 antibody was probed overnight at 4 °C, followed by incubation of secondary antibody tagged with Alexa flour 488 for 1 h at RT and washed with 1X PBS. After washing the excess solution, DNA was counterstained with DAPI, mounted using the antifade medium and imaged immediately using a fluorescence microscope.

### 2.12. PDK1 Inhibitor Treatment 

The well-studied inhibitor of PDK1 GSK 2,334,470 (Tocris), was purchased from Thermofisher Scientific, Waltham, MA USA. After dose optimization, 100 nM concentration was chosen for better inhibition of PDK1 expression in H460 and SKLU cells and their clones. After treatment for 24 h, the cells were harvested, total proteins were extracted, and Western blotting was performed, as described earlier. 

### 2.13. Bioinformatic Analysis

For the initial analysis, a lung adenocarcinoma dataset, the GSE13213 dataset (*n* = 117), was utilized in this study [27]. It comprised 15 patients with a *KRAS* mutation and 102 patients with wild-type variants. A bivariate analysis was conducted to analyze the correlation between *KRAS* and *TIMP*1 expression in two subgroups: *KRAS*-mutated and *KRAS*-wildtype. Another dataset, GSE43580 (*n* = 150) was also utilized in the study [20].Additionally, the RNA-seq gene expression data and somatic mutation data for lung adenocarcinoma (LUAD) samples (*n* = 488) were obtained from the Cancer Genome Atlas (TCGA) dataset (URL: https://portal.gdc.cancer.gov/projects/tcga-luad, accessed on 1 February 2024). Only primary tumor samples with complete mutation annotation and gene expression quantification were included in the analysis. 

### 2.14. Statistical Analysis

Data are presented as means ± standard deviation (SD) for a minimum of three independent experiments. Statistical significance between experimental groups was evaluated using Student’s *t*-test or one-way ANOVA. All analyses were conducted using GraphPad Prism v9.5.1 software. Statistical significance in the figures is indicated as follows: * or # *p* < 0.05; ** *p* < 0.01.

## 3. Results

### 3.1. TIMP-1 Levels Correlate with KRAS Dependency

**Figure 1:**
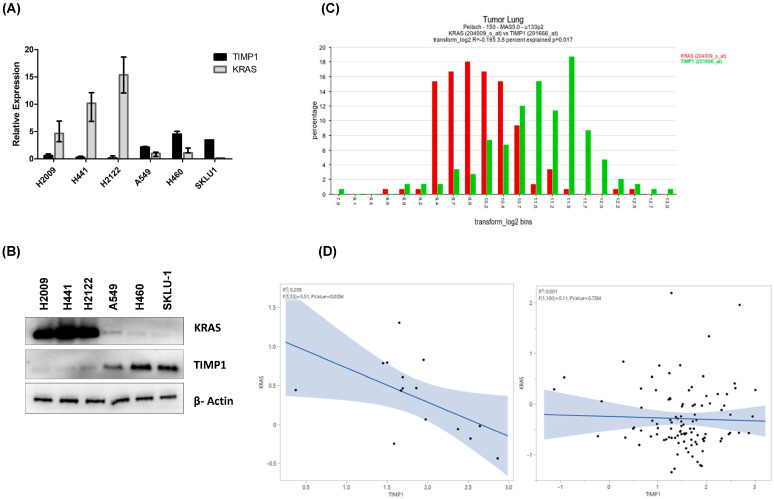
TIMP-1 levels inversely correlate with KRAS dependency: KRAS-dependent NSCLC cell lines H2009, H441, and H2122 and KRAS-independent cells A549, H460, and SKLU-1 were cultured for 48 h. (**A**) *KRAS* and *TIMP*1 mRNA expression in the cell lysates measured by RT-qPCR. (**B**) The KRAS and TIMP-1 protein expression in the cell lysates were detected using Western blotting. (**C**) Showcasing the differential expressions of TIMP1 and KRAS in an independent NSCLC dataset (GSE 43580, *n* = 150 samples) retrieved from an AMC oncogenomics platform. (**D**) The left panel shows that in GSE13213, the KRAS-mutant subgroup showed a negative association between KRAS and TIMP1 expression (R^2^ = 0.29, *p* = 0.03). The right panel shows that in the KRAS-wild type subgroup, the association is negligible (R^2^ = 0.01, *p* = 0.73).

We chose a panel of KRAS-dependent and -independent NSCLC cell lines, as previously defined by Singh et al. [9]. We show that the cell lines they had found to be dependent, and as such, those expressing higher levels of KRAS were expressing low levels of TIMP-1 and vice versa at both RNA and protein levels (Figure 1A,B).

To assess clinical relevance, we explored an independent NSCLC cohort through the AMC OncoGenomics portal (GSE43580), where KRAS and TIMP1 mRNA levels showed an inverse association (Figure 1C) [28]. In another study, GSE13213 (*n* = 117), analysis revealed a negative association in the KRAS-mutant subgroup (R^2^ = 0.29, *p* = 0.03) (Figure 1D left panel), while the KRAS-wildtype subgroup showed no meaningful correlation (R^2^ = 0.01, *p* = 0.73) (Figure 1D right panel) [27]. 

### 3.2. Modulating TIMP-1 Alters KRAS Levels and Affects KRAS Dependency 

**Figure 2:**
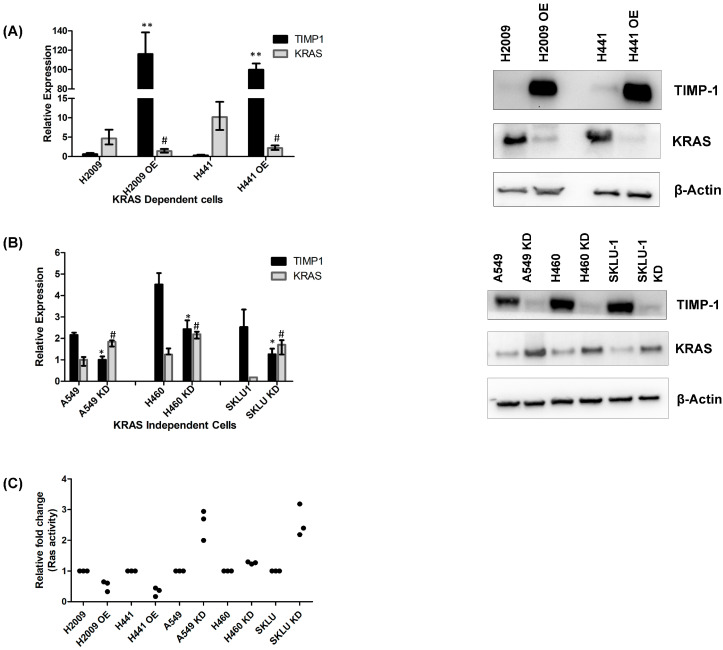
Modulating TIMP-1 alters KRAS levels and affects KRAS dependency: (**A**) The whole cell lysates of KRAS-dependent cells (H2009, H441) and their TIMP-1-overexpressing (OE) clones were used to detect the RNA and protein expression levels. The left panel shows the KRAS and TIMP-1 mRNA levels relative to GAPDH quantified using qPCR, and the right panel shows the protein levels of KRAS and TIMP-1 detected using Western blotting. (**B**) The RNA and protein expression levels of KRAS-independent cells (A549, H460, and SKLU-1) and their TIMP-1 knockdown (KD) clones were studied. The left panel shows the RNA expression levels of KRAS and TIMP-1 relative to GAPDH quantified using qPCR, and the right panel shows the detection of KRAS and TIMP-1 protein levels using Western blotting. (**C**) The fold change in RAS activity of OE and KD clone compared to its respective parental clone is plotted in the graph. ‘*’ (*p* < 0.05) and ‘**’ (*p* < 0.01) indicate the statistically significant differences in TIMP-1 levels of the OE/KD clones from their respective parental clone, and ‘^#^’ indicates the statistically significant differences in KRAS levels of the OE/KD clones from its respective parental clone.

To modulate TIMP-1 levels in these cell lines, we chose two KRAS-dependent cell lines, H2009 and H441, and three KRAS-independent cell lines, A549, SKLU1, and H460. Upon overexpressing TIMP-1 in dependent cell lines, we found that the mRNA and protein levels of KRAS decreased, and on knocking down TIMP-1 in independent cell lines, the KRAS mRNA and protein level increased (Figure 2A,B). 

As knocking down TIMP-1 caused a significant increase in KRAS protein levels, we needed to determine if this KRAS was active in the KD clones of KRAS-independent cell lines. To this end, we carried out an RAS activity assay, and GTP-bound RAS levels were determined by an ELISA assay. This assay measures all the active RAS enzymes, including KRAS. Figure 2C shows RAS-GTP levels decrease upon TIMP-1 overexpression in KRAS-dependent cells. However, upon knocking down TIMP-1, there is an increase in RAS-GTP levels in KRAS-independent cell lines. 

### 3.3. Modulating TIMP-1 Levels Alters Apoptosis upon KRAS Ablation

**Figure 3:**
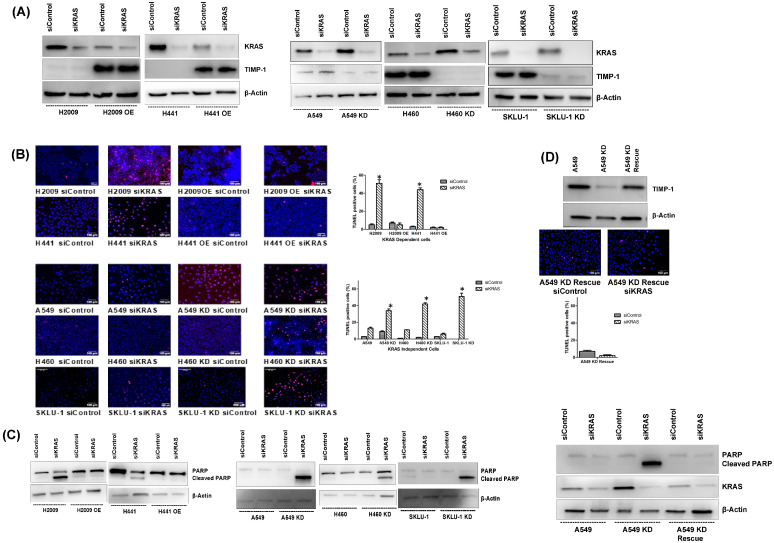
Modulating TIMP-1 levels alters apoptosis upon KRAS ablation: (**A**) Western blot showing the KRAS ablation after siRNA transfection (72 h); (**B**) TUNEL assay performed after silencing KRAS using siRNA transfection. The apoptosis-induced cells show red nuclear fluorescence (TUNEL positive), and the nucleus was counterstained using DAPI (10× magnification). The bar graph shows the quantification of TUNEL apoptosis (%), determined as the proportion of TUNEL-positive cells relative to total DAPI-stained nuclei [*n* = 100 in three different fields]; (**C**) Western blot showing total PARP and cleaved PARP in control and KRAS-silenced cells after 72 h of transfection. siControl represents cells transfected with control siRNA, and siKRAS represents cells transfected with KRAS siRNA. (**D**) KD Rescue—the clone transfected with a plasmid that rescues the knockdown of TIMP-1. Statistically significant differences in the OE/KD clones from their respective parental clones are indicated by ‘*’; *p* < 0.05.

KRAS dependent cell lines undergo apoptosis upon the ablation of *KRAS* using short-hairpin RNA [9,29]. We therefore determined whether TIMP-1 modulation affects KRAS dependency. We first determined that siKRAS does decrease the expression of *KRAS* compared to the control (Figure 3A). We also observed that the TIMP1 levels were not affected when silencing *KRAS*. Interestingly, the overexpression of TIMP-1 in KRAS-dependent cell lines resulted in a decrease in apoptosis upon *KRAS* ablation, as determined by TUNEL assay. Alternatively, we found that knocking down TIMP-1 in KRAS-independent cell lines caused an increase in apoptosis upon *KRAS* ablation (Figure 3B). Substantiating these data, apoptosis responses were assessed by measuring PARP cleavage. During apoptosis, cleavage of the zymogen form of caspases results in their activation. PARP1 is a substrate of activated caspase 3, which cleaves it, causing PARP1′s inactivation. PARP1 is thus unable to repair DNA damage, subsequently leading to apoptosis.

In the KRAS-dependent cell lines, upon KRAS silencing, TIMP-1 OE clone does not undergo PARP cleavage; in contrast, KD TIMP-1 undergoes PARP cleavage in KRAS-independent cells (Figure 3C). A rescue assay was carried out for the A549 KD clone which shows a loss of apoptotic nuclei in the TUNEL assay, and we also find that siKRAS-induced PARP cleavage is lost in the KD clone of A549 with the TIMP-1 rescue plasmid. (Figure 3D). These data indicate that features of KRAS dependency can be altered by TIMP-1 modulation.

### 3.4. Modulating TIMP-1 Levels Alters In Vitro Tumorigenic Profile of NSCLC Cells

**Figure 4:**
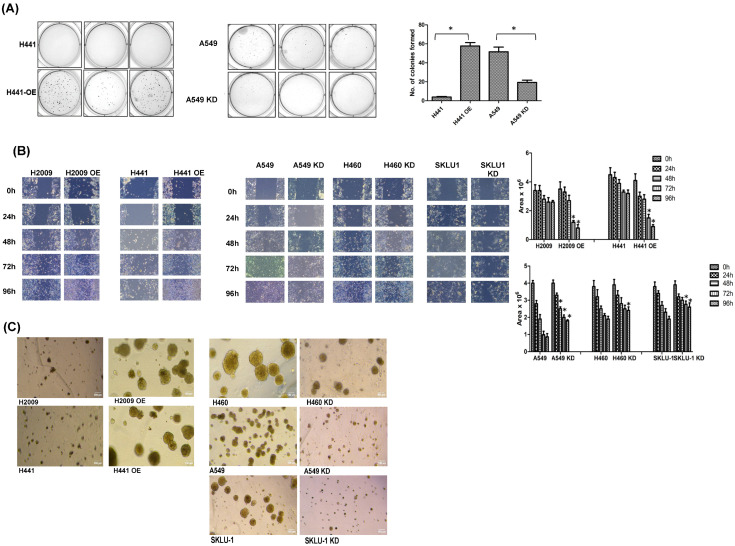
Modulating TIMP1 alters tumorigenicity. (**A**) Anchorage independent growth: The colony formation of KRAS-dependent cells H441 and its TIMP-1 OE clone; KRAS-independent cells A549 and its TIMP-1 KD clone in a soft agar plate (4×). Quantification of colonies formed is plotted with statistically significant differences indicated by ‘*’; *p* < 0.05. (**B**). Wound assay: The migration of cells visualized in KRAS-dependent cells and their TIMP-1 OE cells, and KRAS-independent cells and their KD clones. Quantification of scratch/wound area formed is plotted with statistically significant differences indicated by ‘*’; *p* < 0.05. (**C**) Spheroid assay: Spheroids formed in Matrigel-coated plate visualized under 40× magnification.

To determine any alteration in the tumorigenic profile of the KRAS-dependent and KRAS-independent cell lines upon TIMP-1 modulation, we carried out an anchorage-independent growth assay. The KRAS-dependent cell line H441 showed a negligible number of colonies formed; however, the TIMP-1 OE clones of this cell line showed a remarkable increase in the number of colonies formed (Figure 4A, left panel).

We found that KRAS-independent cell line A549 formed a high number of colonies in soft agar. However, in TIMP-1 KD clones of A549, there is a significant reduction in the number of colonies formed (Figure 4A right panel).

Increases in tumorigenicity and EMT are often associated with increased migratory properties. Overexpression or knockdown of TIMP-1 modulated the migratory properties of NSCLC cell lines, as shown by the wound assay. Figure 4B shows that in the KD clones of KRAS-independent cells, there is a delay in wound closure. In contrast, the OE clones of KRAS-dependent cells show a faster wound closure compared to their respective controls.

We then carried out spheroid formation assays to further confirm tumorigenic potential. The overexpressing clones of TIMP-1 formed larger spheroids that were also increased in number compared to parental or NT cells (Figure 4C left panel). The knockdown clones of TIMP-1 formed smaller and fewer spheroids in relation to the parental cells (Figure 4C right panel). This indicates that tumorigenic properties of the cell line can be altered by modulating TIMP-1.

### 3.5. Differential EMT-Related Marker Expression Is Observed with TIMP-1 Modulation in KRAS-Dependent and -Independent Cells

As KRAS-independence is associated with EMT features, we determined the relative mRNA expression of transcription factors Twist-related protein-1 (TWIST1), ZEB1, and ZEB2. In studies of TIMP1 in EMT, it has been reported that TIMP1 induced TWIST1, ZEB 1, and ZEB2 expression [21,22].

**Figure 5:**
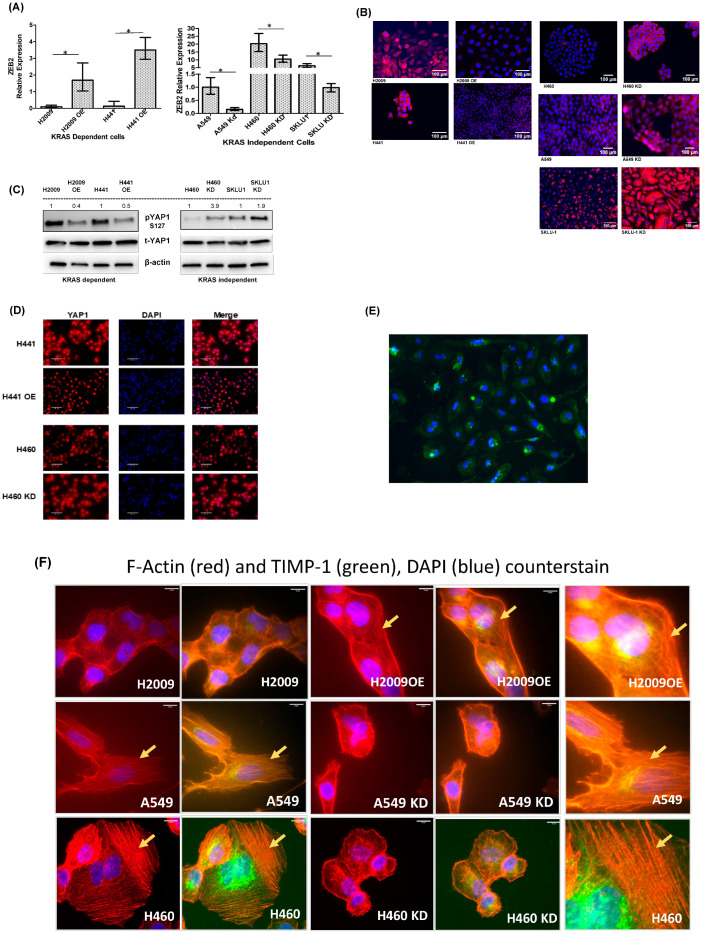
Modulating TIMP-1 levels alters the EMT features: (**A**) ZEB2 mRNA expression levels quantified using q-PCR in KRAS-dependent and KRAS-independent cells. Statistically significant differences are indicated by ‘*’; *p* < 0.05. (**B**) Immunofluorescent staining of the cells stained with E-cadherin antibodies (Red) (100× magnification) counterstained with DAPI, for nuclear staining (blue), 20× magnification. (**C**) Western blot showing overexpression of TIMP-1 activates YAP1, and YAP1 is inactivated TIMP-1 KD clones. (**D**) Immunofluorescent staining of cells tagged with YAP1 antibody (Red) and DAPI for nucleus (Blue). (**E**) Phospho-YAP1 immunofluorescence in H460 cells. (**F**) Immunofluorescent staining of the cells stained with phalloidin (Red); TIMP-1 antibody (green) and counterstained with DAPI (blue), 40× magnification. (**F**) Modulation of TIMP-1 alters the F-actin stress fibers in KRAS-dependent and -independent NSCLC cells, 100× magnification.

We found a higher expression of ZEB2 in KRAS-independent cell lines compared to KRAS-dependent cell lines. However, upon TIMP-1 modulation, the KD clones of A549, H460, and SKLU1 showed a significant decrease in the ZEB2 mRNA relative expression levels. On the other hand, the TIMP-1 OE clone of H2009 and H441 cells showed an increase in the relative expression of ZEB2 (Figure 5A). There was no change in the relative expression of TWIST and ZEB1. 

Previously, we had shown that knocking down TIMP-1 resulted in an increase in E-cadherin levels, as determined by Western blot [24]. We therefore determined the cell staining of E-cadherin in the KRAS-dependent and -independent cell lines and their TIMP-1 modulated clones. As shown in Figure 5B, there is a loss of E-cadherin staining in KRAS-dependent cell lines overexpressing TIMP-1. On the other hand, TIMP-1 knockdown in KRAS-independent cell lines results in the redistribution of E-cadherin.

Recent studies have shown that KRAS-independent cells overcome oncogene addiction by bypassing KRAS and activating YAP1 and/or the RSK-mTOR pathway [30,31]. Multiple studies have shown a role for YAP1 in promoting EMT [32,33,34]. Published reports have shown that KRAS and YAP1 converge to regulate EMT [35]. We therefore determined YAP activation upon TIMP-1 modulation. Phosphorylated YAP1 (s127) is the inactive form and remains confined to the cytoplasm. Upon activation, YAP1 is dephosphorylated and translocated to the nucleus, turning on target genes. In the dependent cells YAP1 is phosphorylated and inactive. When we overexpress TIMP-1, it results in YAP1 activation. 

Figure 5C shows that TIMP-1 overexpression in dependent cell lines results in the activation of YAP1 and vice versa. To confirm the localization of YAP1 in the cells, we carried out immunofluorescence staining in KRAS-dependent, independent, and respective clones. Figure 5D shows that the dependent cell line H441 shows YAP1 staining in the nucleus as well as the cytoplasm. Upon TIMP1 OE, we find that the cytoplasmic staining, i.e., inactive YAP1, is lost, indicating that there is more active YAP1 in TIMP-1 overexpressed clones. On the other hand, we see no inactive cytoplasmic YAP1 in independent H460 cells; however, upon knocking down TIMP1, we can see the presence of YAP1 in the cytoplasm. We also stained H460 cells with pYAP1. Figure 5E shows that this inactive form is confined to the cytoplasm.

During EMT, cells acquire mesenchymal features and migratory potential. The invasive front that develops at the leading edge is dependent on the reorganization of the actin cytoskeleton. There are two main actin forms, globular G-actin, which is the monomer form, and fibrillar F-actin, which is the result of polymerization of G-actin. In adherent cells, upon EMT induction, F-actin polymers are observed as stress fibers [36]. Figure 5F shows that KRAS-independent mesenchymal-like have well-defined stress fibers and upon TIMP-1 KD, cells appear more globular. On the other hand, KRAS-dependent cell line H2009 appears more globular, although stress fibers are more pronounced in the TIMP-1 overexpressing clone of H2009.

### 3.6. Different Signaling Pathways Utilized by KRAS-Dependent and -Independent Cell Lines

**Figure 6:**
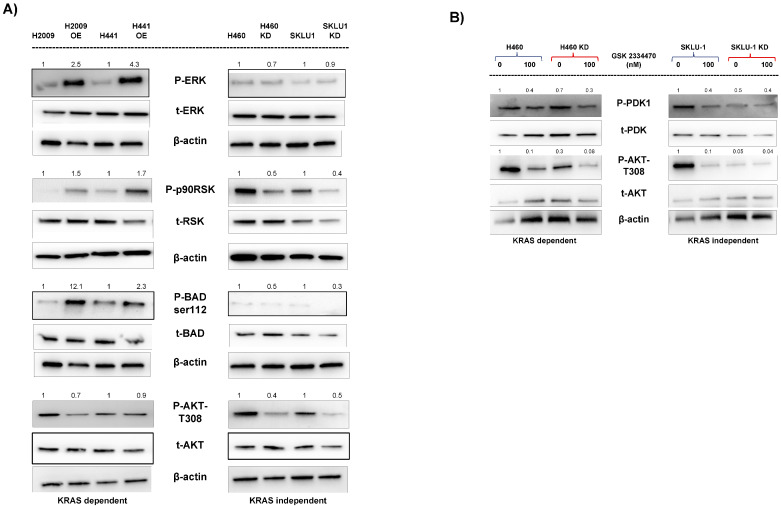
(**A**) Different signaling pathways utilized by KRAS-dependent and -independent cell lines: The protein expression levels of major signaling molecules involved in the RAS-mediated pathway were detected using Western blotting. (**B**) KRAS-independent cells H460, SKLU-1, and their TIMP-1 KD clones were treated with PDK1 inhibitor GSK 2,334,470 (100 nM), and their pAKT expression levels were compared using Western blotting.

Previously, while studying the anti-apoptotic function of TIMP-1, we demonstrated that when we overexpressed TIMP-1 in the KRAS-dependent cell line H2009, there was activation of the MAPK-ERK pathways, as shown by the phospho-activation of the p90RSK—a downstream target of ERK. In KRAS-dependent cell lines, KRAS causes ERK activation [37]. In the present study, we found that overexpressing TIMP-1 in KRAS-dependent cell lines H2009 and H441 led to a robust activation of ERK (Figure 6, left panel) Phospho-p90RSK, the downstream target of pERK, was simultaneously activated. p90RSK is known to phosphorylate the proapoptotic protein BAD at serine 112, causing its retention in the cytoplasm and inhibiting its translocation to the mitochondria. This phosphorylation of S112 inhibits apoptosis in the TIMP-1 OE cells, as previously shown by us [13].

The serine/threonine kinase AKT plays an important role in cell survival and inhibition of apoptosis. We report that pAKT-T308 levels decreased in TIMP-1 OE clones. Since there is a robust increase in pERK, it is possible that this leads to downregulation of pAKT-T308 as crosstalk, and the inhibition of one pathway by another is known to occur [38].

On the other hand, KRAS-independent cells behave somewhat differently in activation of the downstream signaling pathway (Figure 6, right panel). Phospho-ERK is not active in the independent cell lines; rightly so, as ERK activation is considered a feature of KRAS dependency [37,39]. It has recently been shown that KRAS-dependent tumors, such as 95% of PDAC, are driven through the ERK-MAPK pathway [39]. Additionally, they found that drug resistance development leads to the reactivation of the ERK cascade. In an earlier study, Symonds et al. [30] showed that ERK signaling was essential for KRAS-dependent lung carcinoma cells. In another study, it was found that not all mutant KRAS tumors were addicted to KRAS [40]. However, the ones that were addicted showed enrichment of genes upregulated by EGFR, KRAS, or MEK. A recent study has identified TIMP1 as a key factor in maintaining ERK activation in PDAC [41]. However, pAKT- T308 is very active in KRAS-independent cell lines and is totally inactivated upon TIMP-1 knockdown. KRAS independency is gained upon bypassing KRAS via activation of p90RSK and YAP1, both of which we find are active in KRAS-independent cell lines and become inactive upon TIMP-1 knockdown.

An extensive study by Yuan et al. [7] on KRAS mutant cell lines led to two main classifications. The first was KRAS-dependent and also dependent on MAPK/ERK pathway, whereas the second was KRAS-independent but dependent on p90RSK-mTOR via PDK1. Here p90RSK is activated by PDK1, which is downstream of PI3K. Our study finds strong activation of AKT at T308 in independent cell lines, which are known to be phosphorylated by PDK1. Other studies have also documented the importance of PI3/PDK1 in KRAS tumors [42,43]. 

We therefore treated KRAS-independent cell lines and their TIMP-1 KD clone with an inhibitor of PDK1. We found that pPDK1 levels declined following treatment with a concomitant decrease in pAKT T308 levels, as shown in Figure 6B, confirming that T308 phosphorylation occurs via PDK1. We found decreased levels of PDK1 and pPDK1 in the KD clones of TIMP-1 compared to NT clones. Toricelli et al. [14] showed that in melanoma, TIMP-1 activates PDK1 and promotes cell survival. Thus, the reduction in PDK1 levels decreases pAKT T308 upon TIMP-1 knockdown.

**Figure 7:**
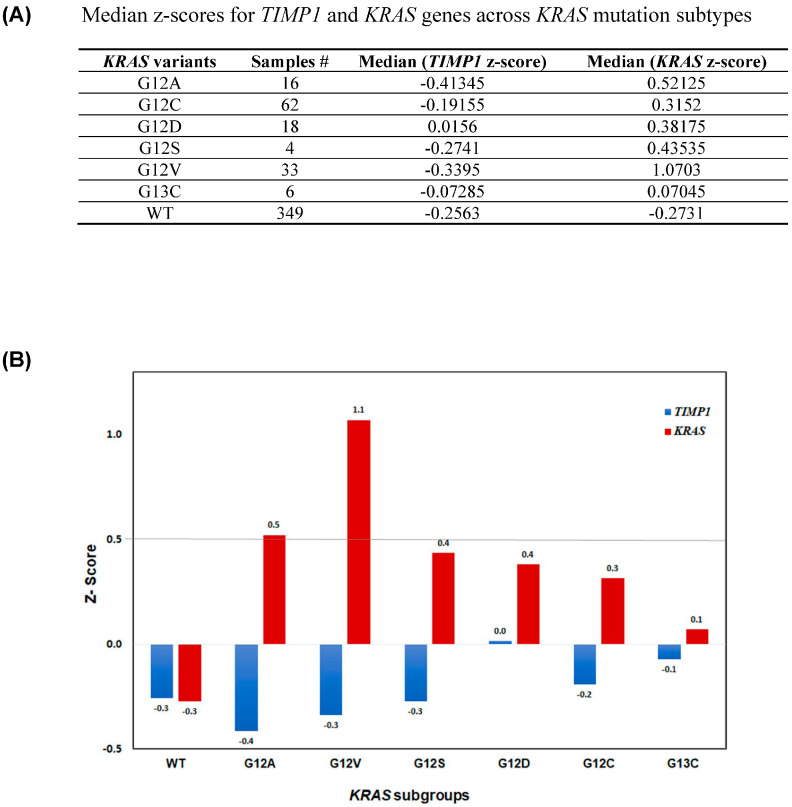
(**A**) TCGA data analysis shows that high *TIMP-1* levels are seen in specific allelic changes, which are associated with *KRAS* independence. (**B**) Bar plot showing the median z-scores for *TIMP-1* and *KRAS* genes across KRAS mutation subtypes (*x*-axis = KRAS mutation groups; *y*-axis = median z-score values).

Finally, to unequivocally confirm the role of TIMP-1 in KRAS dependency, from the TCGA dataset, the median z-scores for *TIMP-1* and *KRAS* gene expression across the different *KRAS* mutation groups are presented in Figure 7A. Graphical representation of the same in Figure 7B shows that low TIMP1 correlates with high KRAS as in variants G12A and G12V (cell lines H2009 and H441). Alternatively, higher TIMP-1 correlates with lower K-RAS levels, as in variants G12S and G12D (cell lines A549 and SKLU-1). 

## 4. Discussion

In the present study, we demonstrate that TIMP-1 expression inversely correlates with level of KRAS expression. High TIMP-1 expression translates to poor prognosis and decreases patient survival. However, the inverse correlation is associated with the oncogene addition function of KRAS, whereby increased KRAS expression is a survival mechanism for tumors addicted to it. 

Beyond its classical function as an inhibitor of MMPs, one of the earliest MMP-independent functions of TIMP-1 was its growth-promoting activity [44]. Indeed, it was first identified as a mitogen. Earlier studies concentrating on its mitogenic properties, documented the relationship between TIMP-1 and RAS. For example, adding recombinant TIMP-1 to osteosarcoma cells activated RAS via phosphorylation of RAF1. It has also been reported that TIMP-1 stimulated the proliferation of human aortic smooth muscle cells by activating RAS [45]. Over the years, several studies have established a more concrete relationship between TIMP-1 and KRAS; e.g., it was shown that knocking down NF-κB in a KRAS lung mouse model decreased TIMP-1 levels, as well as cell proliferation [42]. Other studies have found that TIMP-1 regulated by ERK2 causedhyperproliferation of KRAS pancreatic ductal carcinoma cells [43]. It has been shown that in lung adenocarcinoma, p38α caused increased cell proliferation by promoting the expression of TIMP-1 [46]. Additionally, although KRAS-mutated tumors do not respond to tyrosine kinase inhibitors (TKI), some investigators [47] have shown that KRAS-mutated cells expressing high levels of TIMP-1 responded to TKIs. 

Profiling TIMP-1 in an array of lung adenocarcinoma cell lines, we found that the cell lines depicted as KRAS-independent appeared to express higher levels of TIMP-1 compared to cell lines that were KRAS-dependent. We therefore undertook this study to determine any relationship between TIMP-1 and KRAS. Finding an inverse relationship between TIMP-1 and KRAS expression at both RNA and protein levels, we sought to determine if this held true for patient data. We found clinical relevance in cohorts showing the inverse expression of TIMP-1 and KRAS.

In previous studies, we either used overexpressed or knocked down TIMP-1 in NSCLC cell lines to study various aspects of TIMP-1 functions [24,25]. In the present study, we found that KRAS levels in NSCLC cell lines were altered upon TIMP-1 modulation, again maintaining an inverse relationship such that overexpressing TIMP-1 in a KRAS-dependent cell line decreased KRAS levels in the cell. On the other hand, knocking down TIMP-1 in a KRAS-independent cell line increased KRAS levels. There was a concomitant decrease and increase in RAS-GTP levels, respectively. Singh et al. [9] showed that since KRAS-dependent cell lines are addicted to KRAS, these cells undergo apoptosis upon siRNA knockdown of KRAS. We found that KRAS-dependent cell lines overexpressing TIMP-1 showed reduced apoptosis upon KRAS ablation compared to parental cell lines. Conversely, KRAS-independent cell lines with knocked down TIMP-1 showed increased apoptosis upon KRAS ablation in comparison to parental cell lines. 

Functional assays for tumorigenicity showed that TIMP-1 overexpression in KRAS-dependent cell line allowed it to behave as a KRAS-independent cell line exhibiting a more aggressive phenotype and vice versa. This further establishes a close relationship between KRAS and TIMP-1. Although TIMP1 is known to affect proliferation rate, TIMP1 also has a well-documented anti-apoptotic function [48]. In our earlier studies [13,24,25], KRAS-dependent and -independent cells showed no change in cell proliferation upon TIMP-1 modulation. We have found that TIMP1 strongly affects cell survival. It is possible that in an in vivo TME, both cell proliferation and cell survival are affected by TIMP1. A recent paper [49] shows that upregulated TIMP1 is associated with both proliferation and invasive capacity in colon cancer.

The association of KRAS dependency with epithelial features is now well-established [9,30]. Alternatively, EMT is a crucial phenotype of KRAS-independency [7,9,50]. We found that TIMP-1 modulation altered ZEB2 expression levels in lung cell lines. Several studies have identified ZEB2 as the critical EMT transcription factor whose expression is elevated in a variety of cancers, including NSCLC [51,52,53,54]. We found E-cadherin cell staining to be altered upon TIMP-1 modulation. ZEB2 binds to the promotor of E-cadherin to repress its expression [55,56]. Several studies have shown the upregulation of ZEB2 in NSCLC [53,57]. Recent studies from several labs have demonstrated the transcriptional coactivator YAP1 to bypass KRAS oncogene addiction [31,58]. This study shows that TIMP-1 modulation alters YAP1 activation. TIMP-1 has been shown to promote cell proliferation via YAP/TAZ activation [59]. Interestingly, Shrestha et al. [60] showed that in liposarcoma, the transition of expression from TIMP-4 to TIMP-1 results in aggressive cancer mediated by YAP/TAZ activation. 

KRAS-independent cell lines are known to demonstrate EMT features [7,50]. Several studies have demonstrated that KRAS-independent cells function via YAP1 and/or the RSK-mTOR pathway. The upregulation of YAP1 signaling positively correlated with EMT regulation [35]. Another study [61] found that EGFR-mutated or KRAS-mutated NSCLC were associated with activated YAP1 compared to wild type. Importantly, YAP1 activation in lung adenocarcinoma has been shown to induce ZEB2 expression [62]. Our study shows that TIMP-1 overexpression activates YAP1 and results in the increased expression of ZEB2.

In its MMP-independent tumor-promoting function, TIMP-1 behaves like a cytokine. As such, it is known to bind to specific receptors, causing cell signaling via the MAPK and PI3-Akt pathways, leading to cell survival [63]. Overexpressing TIMP-1 in KRAS-dependent cell lines resulted in several acquired features reminiscent of KRAS-independent cell lines. KRAS levels were altered such that high TIMP-1 allowed these cells to overcome addiction, resulting in lower RAS-GTP bound levels. Furthermore, EMT features, and tumorigenic profile were affected accordingly. However, robust ERK activation is seen in KRAS-dependent cells overexpressing TIMP-1, indicating that seemingly high TIMP-1 expression in KRAS-independent cells must be a downstream effect of the bypass pathways. This is because TIMP-1 levels can modulate KRAS dependency in all aspects except the effector pathway. Thus, the inherent signaling of KRAS via ERK is not altered. This is reinforced by a very recent study showing gene regulation in KRAS-dependent cells occurs predominantly through ERK signaling [39]. Therefore, we further investigated the role of TIMP-1 in KRAS dependency. In an analysis of the TCGA dataset, we identified variant-specific perturbation between KRAS and TIMP-1 expression. Mutant variants that were found to be associated with low TIMP expression were present in the dependent cell lines in our study and high TIMP-1 expression was associated with one of the KRAS-independent cell lines. 

Recent studies have shown the specific allelic mutations of KRAS leading to differential signaling effector engagement (reviewed in [64]). It has been found that KRAS mutant G12D activated the PI3K pathway, whereas G12C activated RAL signaling [65]. Besides biochemical heterogeneity, there is also tissue heterogeneity such that the same allelic change will behave differently in lung versus colon or pancreas [66]. TIMP-1 has a well-documented anti-apoptotic function that has played a critical role in chemoresistance as shown by us and others [17,18,19,67]. In a mouse model of PDAC with a KRAS G12D mutation, D’Costa et al. found that gemcitabine-induced chemoresistance had upregulated TIMP-1 levels [19].

## 5. Conclusions

Oncogene addiction has been documented for KRAS mutants, whereby adenocarcinoma cell lines harboring KRAS mutations have been classified as KRAS-dependent or KRAS-independent [7].This study defines the relationship between KRAS addiction, TIMP-1 expression, and EMT in cancer progression. We show that TIMP-1 levels correlate with KRAS dependency, and that modulating TIMP-1 alters KRAS levels, affecting KRAS-dependency. This modulation also alters apoptosis upon KRAS ablation and alters the tumorigenicity of NSCLC cells. The close relationship between TIMP-1 and EMT in the context of KRAS-dependent and -independent cells is described. Finally, this study identifies different signaling pathways utilized by KRAS-dependent and -independent cell lines. We also analyzed the TCGA dataset to confirm these findings.

Given that high TIMP-1 expression segregates with KRAS-independency, which from our TCGA data analysis shows allelic preference, and the fact that high TIMP-1 in multiple cancers is associated with aggressiveness and poor patient survival, it appears that TIMP-1 holds the potential to be a strong prognostic and predictive marker. Co-therapy with siRNA or a small molecule inhibitor of TIMP-1 thus holds promise for future targeted therapeutic interventions.

## Data Availability

The datasets generated and/or analyzed during the current study are included in this published article. Additionally, publicly available datasets from the AMC Oncogenomics platform and The Cancer Genome Atlas (TCGA) Program were used in this study and can be accessed through their respective repositories.

## Abbreviations

AKT	Ak strain transforming
BAD	Bcl-2-associated death promoter
DMEM	Dulbecco’s Modified Eagle Medium
EGFR	Epidermal Growth Factor Receptor gene
EMT	Epithelial–Mesenchymal Transition
ERK	Extracellular signal-Regulated Kinase
KRAS	Kirsten rat sarcoma viral oncogene homolog
LUAD	Lung Adenocarcinoma
MDCK	Madin-Darby Canine Kidney cells
MMP	Matrix metalloproteinase
NSCLC	Non-Small Cell Lung Carcinoma
PARP	Poly ADP-ribose polymerase
PDAC	Pancreatic Ductal Adenocarcinoma
PMSF	Phenylmethylsulfonyl fluoride
PFA	Para Formaldehyde
RSK	Ribosomal S6 Kinase
SD	Standard Deviation
TIMP-1	Tissue Inhibitor of Metalloproteinase-1
TIMP-1 KD	TIMP-1 Knockdown
TIMP-1 OE	TIMP-1 Over-expressing
TUNEL	Terminal deoxynucleotidyl transferase dUTP nick end labeling
TWIST-1	Twist-associated protein -1
YAP1	yes-associated protein 1
ZEB	Zinc Finger E-Box Binding Homeobox 1 or 2

## References

[1] Suda K., Tomizawa K., Mitsudomi T. (2010). Biological and clinical significance of KRAS mutations in lung cancer: An oncogenic driver that contrasts with EGFR mutation. Cancer Metastasis Rev..

[2] Ding L., Getz G., Wheeler D.A., Mardis E.R., McLellan M.D., Cibulskis K., Sougnez C., Greulich H., Muzny D.M., Morgan M.B. (2008). Somatic mutations affect key pathways in lung adenocarcinoma. Nature.

[3] Tomasini P., Walia P., Labbe C., Jao K., Leighl N.B. (2016). Targeting the KRAS Pathway in Non-Small Cell Lung Cancer. Oncologist.

[4] McCormick F. (2011). Cancer therapy based on oncogene addiction. J. Surg. Oncol..

[5] Salgia R., Pharaon R., Mambetsariev I., Nam A., Sattler M. (2021). The improbable targeted therapy: KRAS as an emerging target in non-small cell lung cancer (NSCLC). Cell Rep. Med..

[6] Riely G.J., Kris M.G., Rosenbaum D., Marks J., Li A., Chitale D.A., Nafa K., Riedel E.R., Hsu M., Pao W. (2008). Frequency and distinctive spectrum of KRAS mutations in never smokers with lung adenocarcinoma. Clin. Cancer Res..

[7] Yuan T.L., Amzallag A., Bagni R., Yi M., Afghani S., Burgan W., Fer N., Strathern L.A., Powell K., Smith B. (2018). Differential Effector Engagement by Oncogenic KRAS. Cell Rep..

[8] Weinstein I.B. (2002). Cancer. Addiction to oncogenes—The Achilles heal of cancer. Science.

[9] Singh A., Greninger P., Rhodes D., Koopman L., Violette S., Bardeesy N., Settleman J. (2009). A gene expression signature associated with “K-Ras addiction” reveals regulators of EMT and tumor cell survival. Cancer Cell.

[10] Kalluri R., Weinberg R.A. (2009). The basics of epithelial-mesenchymal transition. J. Clin. Investig..

[11] Brew K., Nagase H. (2010). The tissue inhibitors of metalloproteinases (TIMPs): An ancient family with structural and functional diversity. Biochim. Biophys. Acta.

[12] Jackson H.W., Defamie V., Waterhouse P., Khokha R. (2017). TIMPs: Versatile extracellular regulators in cancer. Nat. Rev. Cancer.

[13] Nalluri S., Ghoshal-Gupta S., Kutiyanawalla A., Gayatri S., Lee B.R., Jiwani S., Rojiani A.M., Rojiani M.V. (2015). TIMP-1 Inhibits Apoptosis in Lung Adenocarcinoma Cells via Interaction with Bcl-2. PLoS ONE.

[14] Toricelli M., Melo F.H.M., Hunger A., Zanatta D., Strauss B.E., Jasiulionis M.G. (2017). Timp1 Promotes Cell Survival by Activating the PDK1 Signaling Pathway in Melanoma. Cancers.

[15] Liu X.W., Taube M.E., Jung K.K., Dong Z., Lee Y.J., Roshy S., Sloane B.F., Fridman R., Kim H.R. (2005). Tissue inhibitor of metalloproteinase-1 protects human breast epithelial cells from extrinsic cell death: A potential oncogenic activity of tissue inhibitor of metalloproteinase-1. Cancer Res..

[16] Lambert E., Bridoux L., Devy J., Dasse E., Sowa M.L., Duca L., Hornebeck W., Martiny L., Petitfrere-Charpentier E. (2009). TIMP-1 binding to proMMP-9/CD44 complex localized at the cell surface promotes erythroid cell survival. Int. J. Biochem. Cell Biol..

[17] Xiao W., Wang L., Howard J., Kolhe R., Rojiani A.M., Rojiani M.V. (2019). TIMP-1-Mediated Chemoresistance via Induction of IL-6 in NSCLC. Cancers.

[18] Fu Z.Y., Lv J.H., Ma C.Y., Yang D.P., Wang T. (2011). Tissue inhibitor of metalloproteinase-1 decreased chemosensitivity of MDA-435 breast cancer cells to chemotherapeutic drugs through the PI3K/AKT/NF-small ka, CyrillicB pathway. Biomed. Pharmacother..

[19] D’Costa Z., Jones K., Azad A., van Stiphout R., Lim S.Y., Gomes A.L., Kinchesh P., Smart S.C., Gillies McKenna W., Buffa F.M. (2017). Gemcitabine-Induced TIMP1 Attenuates Therapy Response and Promotes Tumor Growth and Liver Metastasis in Pancreatic Cancer. Cancer Res..

[20] Sa Y., Li C., Li H., Guo H. (2015). TIMP-1 Induces alpha-Smooth Muscle Actin in Fibroblasts to Promote Urethral Scar Formation. Cell Physiol. Biochem..

[21] Jung Y.S., Liu X.W., Chirco R., Warner R.B., Fridman R., Kim H.R. (2012). TIMP-1 induces an EMT-like phenotypic conversion in MDCK cells independent of its MMP-inhibitory domain. PLoS ONE.

[22] D’Angelo R.C., Liu X.W., Najy A.J., Jung Y.S., Won J., Chai K.X., Fridman R., Kim H.R. (2014). TIMP-1 via TWIST1 induces EMT phenotypes in human breast epithelial cells. Mol. Cancer Res..

[23] Mathias R.A., Wang B., Ji H., Kapp E.A., Moritz R.L., Zhu H.J., Simpson R.J. (2009). Secretome-based proteomic profiling of Ras-transformed MDCK cells reveals extracellular modulators of epithelial-mesenchymal transition. J. Proteome Res..

[24] Ghoshal-Gupta S., Kutiyanawalla A., Lee B.R., Ojha J., Nurani A., Mondal A.K., Kolhe R., Rojiani A.M., Rojiani M.V. (2018). TIMP-1 downregulation modulates miR-125a-5p expression and triggers the apoptotic pathway. Oncotarget.

[25] Rojiani M.V., Ghoshal-Gupta S., Kutiyanawalla A., Mathur S., Rojiani A.M. (2015). TIMP-1 overexpression in lung carcinoma enhances tumor kinetics and angiogenesis in brain metastasis. J. Neuropathol. Exp. Neurol..

[26] Xiao W., Ahluwalia P., Wang L., Howard J., Kolhe R., Rojiani A.M., Rojiani M.V. (2022). TIMP-1 Dependent Modulation of Metabolic Profiles Impacts Chemoresistance in NSCLC. Cells.

[27] Tomida S., Takeuchi T., Shimada Y., Arima C., Matsuo K., Mitsudomi T., Yatabe Y., Takahashi T. (2009). Relapse-related molecular signature in lung adenocarcinomas identifies patients with dismal prognosis. J. Clin. Oncol..

[28] Tarca A.L., Bhatti G., Romero R. (2013). A comparison of gene set analysis methods in terms of sensitivity, prioritization and specificity. PLoS ONE.

[29] Pettazzoni P., Viale A., Shah P., Carugo A., Ying H., Wang H., Genovese G., Seth S., Minelli R., Green T. (2015). Genetic events that limit the efficacy of MEK and RTK inhibitor therapies in a mouse model of KRAS-driven pancreatic cancer. Cancer Res..

[30] Adachi Y., Kimura R., Hirade K., Ebi H. (2021). Escaping KRAS: Gaining Autonomy and Resistance to KRAS Inhibition in KRAS Mutant Cancers. Cancers.

[31] Kapoor A., Yao W., Ying H., Hua S., Liewen A., Wang Q., Zhong Y., Wu C.J., Sadanandam A., Hu B. (2014). Yap1 activation enables bypass of oncogenic Kras addiction in pancreatic cancer. Cell.

[32] Yu M., Chen Y., Li X., Yang R., Zhang L., Huangfu L., Zheng N., Zhao X., Lv L., Hong Y. (2018). YAP1 contributes to NSCLC invasion and migration by promoting Slug transcription via the transcription co-factor TEAD. Cell Death Dis..

[33] Chen N., Golczer G., Ghose S., Lin B., Langenbucher A., Webb J., Bhanot H., Abt N.B., Lin D., Varvares M. (2022). YAP1 maintains active chromatin state in head and neck squamous cell carcinomas that promotes tumorigenesis through cooperation with BRD4. Cell Rep..

[34] Guo Z., Zhou K., Wang Q., Huang Y., Ji J., Peng Y., Zhang X., Zheng T., Zhang Z., Chong D. (2021). The transcription factor RUNX2 fuels YAP1 signaling and gastric cancer tumorigenesis. Cancer Sci..

[35] Shao D.D., Xue W., Krall E.B., Bhutkar A., Piccioni F., Wang X., Schinzel A.C., Sood S., Rosenbluh J., Kim J.W. (2014). KRAS and YAP1 converge to regulate EMT and tumor survival. Cell.

[36] Schneider D., Baronsky T., Pietuch A., Rother J., Oelkers M., Fichtner D., Wedlich D., Janshoff A. (2013). Tension monitoring during epithelial-to-mesenchymal transition links the switch of phenotype to expression of moesin and cadherins in NMuMG cells. PLoS ONE.

[37] Symonds J.M., Ohm A.M., Tan A.C., Reyland M.E. (2016). PKCdelta regulates integrin alphaVbeta3 expression and transformed growth of K-ras dependent lung cancer cells. Oncotarget.

[38] Mendoza M.C., Er E.E., Blenis J. (2011). The Ras-ERK and PI3K-mTOR pathways: Cross-talk and compensation. Trends Biochem. Sci..

[39] Klomp J.E., Diehl J.N., Klomp J.A., Edwards A.C., Yang R., Morales A.J., Taylor K.E., Drizyte-Miller K., Bryant K.L., Schaefer A. (2024). Determining the ERK-regulated phosphoproteome driving KRAS-mutant cancer. Science.

[40] Tyc K.M., Kazi A., Ranjan A., Wang R., Sebti S.M. (2023). Novel mutant KRAS addiction signature predicts response to the combination of ERBB and MEK inhibitors in lung and pancreatic cancers. iScience.

[41] Wang C.A., Hou Y.C., Hong Y.K., Tai Y.J., Shen C., Hou P.C., Fu J.L., Wu C.L., Cheng S.M., Hwang D.Y. (2025). Intercellular TIMP-1-CD63 signaling directs the evolution of immune escape and metastasis in KRAS-mutated pancreatic cancer cells. Mol. Cancer.

[42] Wang G.M., Wong H.Y., Konishi H., Blair B.G., Abukhdeir A.M., Gustin J.P., Rosen D.M., Denmeade S.R., Rasheed Z., Matsui W. (2013). Single copies of mutant KRAS and mutant PIK3CA cooperate in immortalized human epithelial cells to induce tumor formation. Cancer Res..

[43] Eser S., Reiff N., Messer M., Seidler B., Gottschalk K., Dobler M., Hieber M., Arbeiter A., Klein S., Kong B. (2013). Selective requirement of PI3K/PDK1 signaling for Kras oncogene-driven pancreatic cell plasticity and cancer. Cancer Cell.

[44] Jiang Y., Goldberg I.D., Shi Y.E. (2002). Complex roles of tissue inhibitors of metalloproteinases in cancer. Oncogene.

[45] Akahane T., Akahane M., Shah A., Thorgeirsson U.P. (2004). TIMP-1 stimulates proliferation of human aortic smooth muscle cells and Ras effector pathways. Biochem. Biophys Res. Commun..

[46] Vitos-Faleato J., Real S.M., Gutierrez-Prat N., Villanueva A., Llonch E., Drosten M., Barbacid M., Nebreda A.R. (2020). Requirement for epithelial p38alpha in KRAS-driven lung tumor progression. Proc. Natl. Acad. Sci. USA.

[47] Tarpgaard L.S., Orum-Madsen M.S., Christensen I.J., Nordgaard C., Noer J., Guren T.K., Glimelius B., Sorbye H., Ikdahl T., Kure E.H. (2016). TIMP-1 is under regulation of the EGF signaling axis and promotes an aggressive phenotype in KRAS-mutated colorectal cancer cells: A potential novel approach to the treatment of metastatic colorectal cancer. Oncotarget.

[48] Chirco R., Liu X.W., Jung K.K., Kim H.R. (2006). Novel functions of TIMPs in cell signaling. Cancer Metastasis Rev..

[49] Ma B., Ueda H., Okamoto K., Bando M., Fujimoto S., Okada Y., Kawaguchi T., Wada H., Miyamoto H., Shimada M. (2022). TIMP1 promotes cell proliferation and invasion capability of right-sided colon cancers via the FAK/Akt signaling pathway. Cancer Sci..

[50] Collisson E.A., Sadanandam A., Olson P., Gibb W.J., Truitt M., Gu S., Cooc J., Weinkle J., Kim G.E., Jakkula L. (2011). Subtypes of pancreatic ductal adenocarcinoma and their differing responses to therapy. Nat. Med..

[51] Rosivatz E., Becker I., Specht K., Fricke E., Luber B., Busch R., Hofler H., Becker K.F. (2002). Differential expression of the epithelial-mesenchymal transition regulators snail, SIP1, and twist in gastric cancer. Am. J. Pathol..

[52] Zhu X., Wei L., Bai Y., Wu S., Han S. (2017). FoxC1 promotes epithelial-mesenchymal transition through PBX1 dependent transactivation of ZEB2 in esophageal cancer. Am. J. Cancer Res..

[53] Shi Z.M., Wang L., Shen H., Jiang C.F., Ge X., Li D.M., Wen Y.Y., Sun H.R., Pan M.H., Li W. (2017). Downregulation of miR-218 contributes to epithelial-mesenchymal transition and tumor metastasis in lung cancer by targeting Slug/ZEB2 signaling. Oncogene.

[54] You J., Li Y., Fang N., Liu B., Zu L., Chang R., Li X., Zhou Q. (2014). MiR-132 suppresses the migration and invasion of lung cancer cells via targeting the EMT regulator ZEB2. PLoS ONE.

[55] Vandewalle C., Comijn J., De Craene B., Vermassen P., Bruyneel E., Andersen H., Tulchinsky E., Van Roy F., Berx G. (2005). SIP1/ZEB2 induces EMT by repressing genes of different epithelial cell-cell junctions. Nucleic Acids Res..

[56] Comijn J., Berx G., Vermassen P., Verschueren K., van Grunsven L., Bruyneel E., Mareel M., Huylebroeck D., van Roy F. (2001). The two-handed E box binding zinc finger protein SIP1 downregulates E-cadherin and induces invasion. Mol. Cell.

[57] Wu D.M., Zhang T., Liu Y.B., Deng S.H., Han R., Liu T., Li J., Xu Y. (2019). The PAX6-ZEB2 axis promotes metastasis and cisplatin resistance in non-small cell lung cancer through PI3K/AKT signaling. Cell Death Dis..

[58] Tu B., Yao J., Ferri-Borgogno S., Zhao J., Chen S., Wang Q., Yan L., Zhou X., Zhu C., Bang S. (2019). YAP1 oncogene is a context-specific driver for pancreatic ductal adenocarcinoma. JCI Insight.

[59] Ando T., Charindra D., Shrestha M., Umehara H., Ogawa I., Miyauchi M., Takata T. (2018). Tissue inhibitor of metalloproteinase-1 promotes cell proliferation through YAP/TAZ activation in cancer. Oncogene.

[60] Shrestha M., Ando T., Chea C., Sakamoto S., Nishisaka T., Ogawa I., Miyauchi M., Takata T. (2019). The transition of tissue inhibitor of metalloproteinases from −4 to −1 induces aggressive behavior and poor patient survival in dedifferentiated liposarcoma via YAP/TAZ activation. Carcinogenesis.

[61] Cheng H., Zhang Z., Rodriguez-Barrueco R., Borczuk A., Liu H., Yu J., Silva J.M., Cheng S.K., Perez-Soler R., Halmos B. (2016). Functional genomics screen identifies YAP1 as a key determinant to enhance treatment sensitivity in lung cancer cells. Oncotarget.

[62] Gao Y., Zhang W., Han X., Li F., Wang X., Wang R., Fang Z., Tong X., Yao S., Li F. (2014). YAP inhibits squamous transdifferentiation of Lkb1-deficient lung adenocarcinoma through ZEB2-dependent DNp63 repression. Nat. Commun..

[63] Ries C. (2014). Cytokine functions of TIMP-1. Cell. Mol. Life Sci..

[64] Huang L., Guo Z., Wang F., Fu L. (2021). KRAS mutation: From undruggable to druggable in cancer. Signal Transduct. Target. Ther..

[65] Ihle N.T., Byers L.A., Kim E.S., Saintigny P., Lee J.J., Blumenschein G.R., Tsao A., Liu S., Larsen J.E., Wang J. (2012). Effect of KRAS oncogene substitutions on protein behavior: Implications for signaling and clinical outcome. J. Natl. Cancer Inst..

[66] Tripathi P., Kumari R., Pathak R. (2024). Drugging the undruggable: Advances in targeting KRAS signaling in solid tumors. Int. Rev. Cell Mol. Biol..

[67] Davidsen M.L., Wurtz S.O., Romer M.U., Sorensen N.M., Johansen S.K., Christensen I.J., Larsen J.K., Offenberg H., Brunner N., Lademann U. (2006). TIMP-1 gene deficiency increases tumour cell sensitivity to chemotherapy-induced apoptosis. Br. J. Cancer.

